# Protocol for aerial trapping and analyses of candidate pheromone compounds released by moths via gas chromatography-mass spectrometry

**DOI:** 10.1016/j.xpro.2024.103293

**Published:** 2024-09-05

**Authors:** Sagnik Ghosh, Antonio Palazzo, Jérémy Gévar, Philippe Lucas, Abhishek Chatterjee

**Affiliations:** 1 Institute of Ecology and Environmental Sciences of Paris (iEES-*Paris*), INRAE, Sorbonne University, CNRS, IRD, UPEC, Université Paris Cité, 78026 Versailles, France

**Keywords:** Neuroscience, Behavior, Mass Spectrometry, Environmental sciences

## Abstract

Detection of pheromones is pivotal to chemical ecology and agronomy; however, analytic detection of the volatile pheromone components from odorized air is highly challenging. Here, we introduce a protocol for the detection of airborne pheromones from female moths, which are key models for chemosensory studies. We describe a step-by-step guide from pheromone collection to quantitative estimation of pheromone components. We also detail procedures for gas chromatography-mass spectrometry (GC-MS) analysis. This protocol has potential applications beyond chemosensory research, particularly in environmental chemistry.

For complete details on the use and execution of this protocol, please refer to Ghosh et al.^1^

## Highlights

• Instructions for implementing suitable biological conditions for pheromone release
• Guide to collecting airborne pheromone components using a Tenax TA cartridge
• Steps for using a thermal desorption unit to inject samples into the GC-MS
• Inject standards to generate a standard curve and estimate the retention indices

## Abstract (editor highlights)

Publisher’s note: Undertaking any experimental protocol requires adherence to local institutional guidelines for laboratory safety and ethics.

## Graphical abstract

**Figure:**
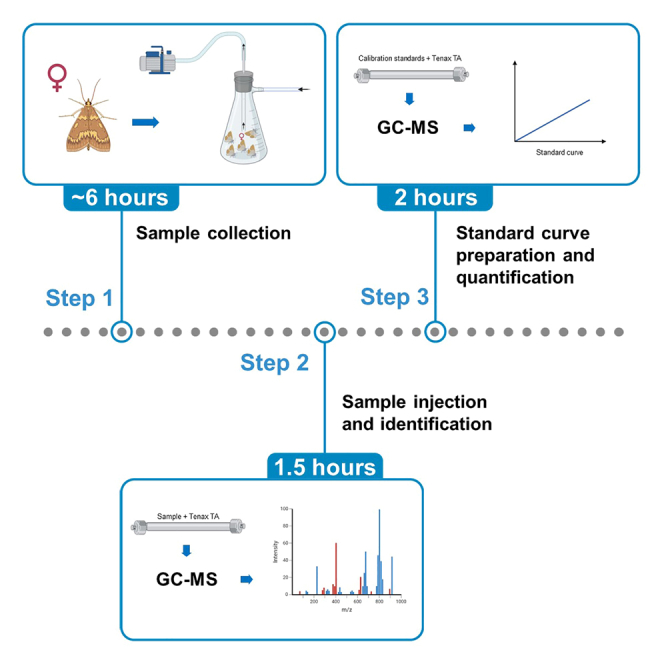

## Before you begin

**Figure 1:**
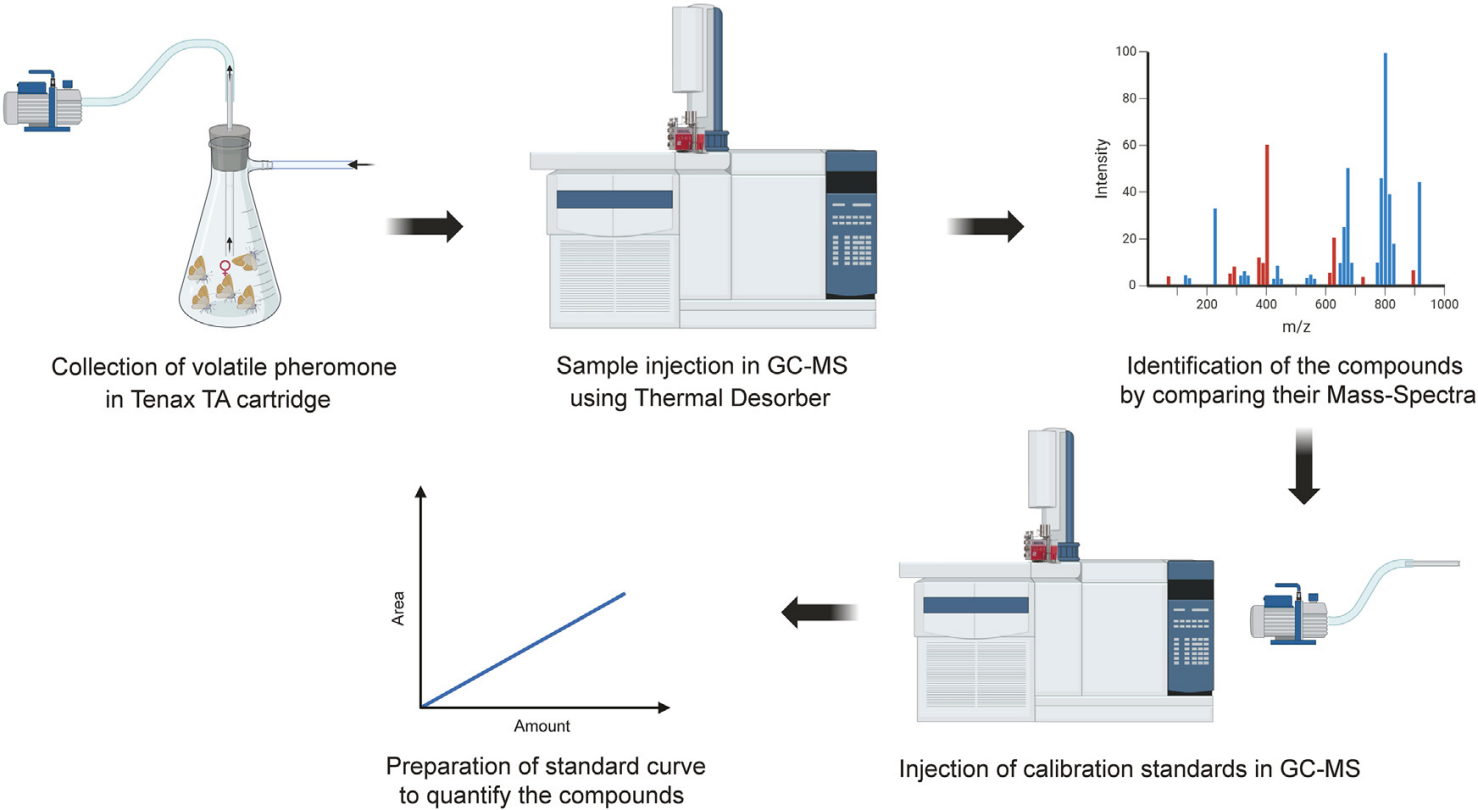
A step-by-step schematic of the collection, identification and characterization of the volatile pheromone components released by female moths

The protocol below (Figure 1) describes the process of collecting the airborne candidate pheromone compounds and identifying them under GC-MS.

### Moth rearing

**Timing: variable**

1. Rear moth larvae using a semi-artificial diet^2^ in incubators with precisely controlled temperature, light cycle and relative humidity.
2. When in the pupal stage, separate the males and females and keep them separate.
3. A few days before emergence, transfer the pupas to incubators having 23°C temperature, 60% relative humidity, and 16 h:8 h of Light-Dark cycles, which mimics the summer conditions in temperate countries.
4. Upon emergence, keep the young moths at 22°C with 12% sugar water.

### Institutional permissions

5. The European Union directive 182 2010/63/EU allows manipulation of moths without formal ethical measures. Similar country-specific regulations should be verified before working with moths. The users of this protocol should take proper measures during handling the moths in order to minimize their discomfort.

### Conditioning of the Tenax TA cartridge

**Timing: 3 h**

6. Condition the Tenax TA cartridge to be used for pheromone collection for 3 h at 320°C using a specific cartridge conditioner (Figure S1A).

**CRITICAL:** Correctly insert the cartridge into the conditioner, with the ridged section of the cartridge facing upward.

### Assemble insect volatile collection setup

**Timing: 30 min**

7. Connect the conditioned cartridge with the outlet of a Drechsel bottle head using Tygon PVC tubes. Use parafilm to make the connections airtight.
8. Connect the inlet of the same Drechsel bottle head to a compressed air system fitted with a flow regulator, humidifier and charcoal filter.

***Note:*** Properly clean every glassware using acetone and hexane followed by heating them at 150°C for 2–3 h to avoid any odor contamination.

## Key resources table

**Table:** 

REAGENT or RESOURCE	SOURCE	IDENTIFIER
**Chemicals, peptides, and recombinant proteins**		

n-Hexane, 99% purity	CARLO ERBA Reagents	110-54-3
n-Alkane standard solution (C8-C20)	Supelco	04070
(Z)-9-Tetradecen-1-ol acetate (Z9-14Ac), >99% purity	Pherobank	16725-53-4
(E)11-Tetradecen-1-ol acetate (E11-14Ac), >99% purity	Pherobank	33189-72-9
(Z)-11-Tetradecen-1-ol acetate (Z11-14Ac), >99% purity	Pherobank	20711-10-8
(Z, E)-9,11-Tetradecadien-1-ol acetate (Z9,E11-14Ac), >96% purity	Pherobank	50767-79-8

**Experimental models: Organisms/strains**		

Adult female *Spodoptera littoralis*	INRAE Versailles	N/A

**Software and algorithms**		

Agilent MassHunter	Agilent, USA	https://www.agilent.com/en/promotions/masshunter-mass-spec
NIST Mass Spectral library	National Institute of Standards and Technology, USA	Tandem Mass Spectral Library | NIST

**Other**		

GC-MS system	Agilent, USA	8890-5977B
Thermal Desorber TD 3.5+	Gerstel, Germany	N/A
HP-5ms Capillary column	Agilent, USA	N/A
Tenax TA cartridges	Gerstel, Germany	N/A
APK1200 conditioner	KNR, South Korea	N/A
Drechsel bottle head	Fisher Scientific, UK	N/A
500 mL Erlenmeyer (conical) flask	Amazon	N/A
GilAir Plus Pump	Sensidyne, USA	N/A
Tygon PVC tubes (inner diameter 6 mm)	Saint-Gobain, France	N/A
Micropipette 1–10 μL	Dutscher	711321
Mini vortexer	Amazon	N/A

## Materials and equipment

**Table:** Calibration Standard Solution (1 mg/L)

Reagent	Final concentration	Amount
Z9-14Ac	0.1 μg/μL	10 μL
E11-14Ac	0.1 μg/μL	10 μL
Z11-14Ac	0.1 μg/μL	10 μL
Z9, E11-14Ac	0.1 μg/μL	10 μL
n-Hexane	Solvent	960 μL
**Total**	**1 mg/L**	**1 mL**

***Note:*** The standard solution can be stored at −20°C for upto 1 month.

### Reagent safety statements

**CRITICAL:** Hexane is a flammable and toxic liquid if inhaled, ingested, or applied to the skin. Proper personal protective equipment (i.e., gloves, goggles and lab coat) and engineering controls (i.e., chemical fume hood) should be used while handling it. Please refer to the Hexane safety data sheet for further details.

## Step-by-step method details

### Aerial collection of insect volatile

**Timing: 5 h**

This step describes the process of collecting airborne candidate pheromone compounds released by a female moth.

**Figure 2:**
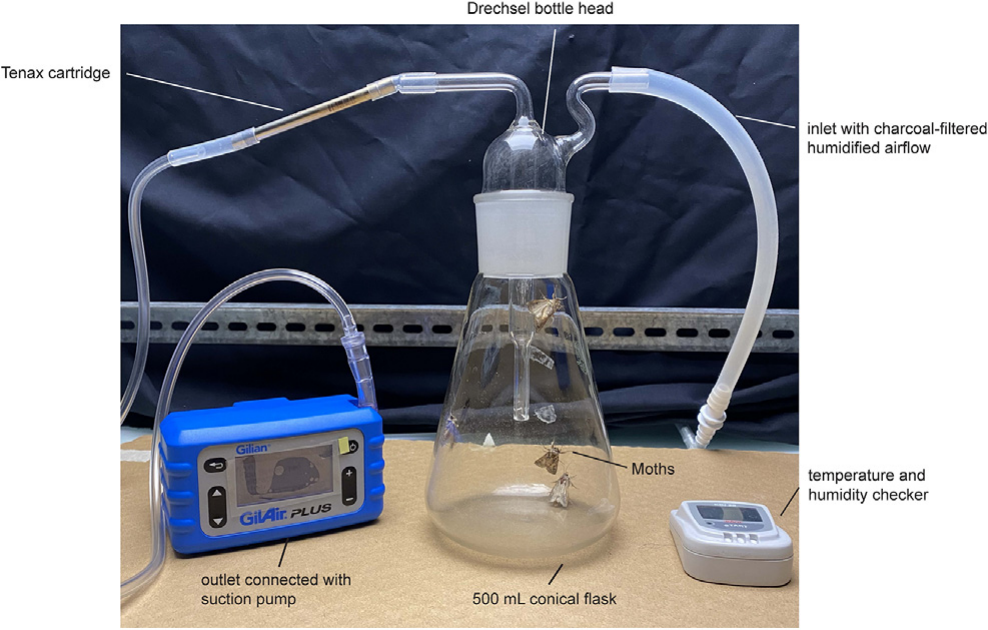
An example setup used to collect airborne pheromones released by moths Notably, the use of silicon connections should be minimized as much as possible.

1. Insect volatile collection method:
  a. Put around 10–12 adult virgin female moths of around 2–3 days of age in a 500 mL conical flask. We used the *Spodoptera littoralis* French strain for our experiment.
    ***Note:*** Make sure to entrain the young female moths to a 16 h:8 h light-dark cycle (in our case, light on at 7 pm, light off at 11 am) for 2 days before the experiment.
  b. Close the flask containing female moths with the Drechsel bottle head whose outlet is connected to the Tenax TA cartridge (Figure 2).
  c. Put the whole setup in a dark arena with a temperature of 22°C and humidity around 60%–70%.
  d. Connect the outlet of the Tenax TA cartridge to a suction pump and set the flow rate of the pump to 200 mL/min.
    ***Note:*** Make sure that the Tenax TA cartridge has been connected in the right orientation with the arrow tip or the unridged end of the cartridge facing the pump.
  e. Start the charcoal-filtered, humidified, inlet airflow and regulate the airflow to 200 mL/min.
  f. Start the pump and let the setup in the same condition for 5 h in order to get the candidate pheromone compounds adsorbed to the cartridge.
    **CRITICAL:** The pheromone release rate peaks around the middle of the subjective night for the nocturnal moths. So, for maximum efficiency, the pheromone collection should be started around the start of their subjective night, depending on the entrainment conditions. We used zeitgeber time (ZT) 16–21 for collecting pheromones of *S. littoralis* females.
    **Pause point:** Samples collected in a cartridge can be stored at −20°C for up to 1 month before injecting into GC-MS. Make sure that the cartridge is closed hermetically with the caps during the storage.

### Sample injection and identification of the candidate pheromone compounds

**Timing: 1 h**

This step describes the process of injecting the sample obtained into the GC-MS along with identifying the compounds present in the pheromone samples, detected via GC-MS.

2. GC-MS method:
  a. Use a Thermal Desorption Unit (TD 3.5+) for injecting the sample adsorbed by Tenax TA cartridges into GC-MS.
    **CRITICAL:** For injecting volatile samples using Tenax TA, the Thermal Desorption Unit is crucial and cannot be substituted.
  b. Desorb the Tenax TA cartridge in the splitless mode at an initial temperature of 30°C for 20 min, followed by an increase to 280°C at a ramp rate of 60°C/min with a final hold of 5 min at that temperature (Figure S2A).
  c. Inject the samples with the programmable temperature vaporizing inlet (CIS/PTV), which should earlier be kept at −40°C during the desorption phase, but then heated to 280°C at a ramp rate of 12°C/s with a final hold for 5 min during the injection (Figure S2B).
  d. For chromatographic separation:
    i. Employ a (5%-phenyl)-methylpolysiloxane nonpolar column capillary GC column (Agilent Technologies HP-5ms column, 30 m length × 0,25 mm internal diameter, 0.25 μm film thickness) with helium as the carrier gas.
      ***Note:*** The carrier gas should have a constant flow rate of 1 mL/min.
    ii. Use an initial oven temperature of 35°C, then increase it to 290°C at a ramp rate of 8°C/min, with a final hold of 10 min at that temperature (Figure S2C).
  e. For the Mass-Spectrometry:
    i. Set the temperature of the transfer line to 250°C, the ionization source to 230°C, and the quadrupole mass analyzer to 150°C.
    ii. Ionize the sample in the electron impact mode (70 eV) and collect mass spectral data in the full scan mode with a mass range of 30–400 m/z.
      ***Note:*** For our analysis, the total run-time of the GC-MS is 42.78 min.
3. Identification of the samples:
  a. Open the obtained data in MassHunter Qualitative software.
  b. Zoom and double-click on each of the major peaks of interest to obtain the mass spectrum.
  c. Right click on the spectrum and select “Search using NIST MS Program.”
  d. Once the NIST library is open, compare your mass spectrum with the reference spectrum proposed by the library (Figure S3A). Also, check the match value obtained (Figure S3B).
  e. Identify your compounds of interest guided by published literatures and note down their respective retention times (RT).

***Note:*** For an ideal identification, the match value should be above 900. Also, the calculated retention index (described below) for each compound of interest should match with the one mentioned in the library.

### Preparation and injection of calibration standards

**Timing: 1.5 h**

This step provides with the detailed procedure of preparing the standard solutions, spiking the cartridges with those solutions and injecting them into GC-MS.

4. Preparation of the standard solutions:
  a. Weigh approximately 1 mg of each analytical standard, and dissolve them in the proper amount of n-Hexane in order to achieve a concentration of 10 μg/μL.
  b. Make a 100 times dilution of each of the 10 μg/μL standard solutions using n-Hexane to get a concentration of 0.1 μg/μL.
    ***Note:*** After each dilution, shake the final solution vigorously using a vortexer.
  c. Prepare a working solution of the analytical standards by mixing 10 μL of each of the individual 0.1 μg/μL standard solutions and making up the final volume up to 1000 μL using n-hexane.
    ***Note:*** In our case, we had 4 analytical standards, so we have added 960 μL of n-Hexane to the 40 μL of mix in order to obtain 1 mL of 1 mg/L final working solution.
    **CRITICAL:** Appropriate concentration of the working solution of the standards will vary depending on the experiment and will need to be individually determined and adjusted.
5. Injecting the standards into GC-MS:
  a. Use 1,10, 20, 50 and 200 ng of the final working solution of the standards (1 mg/L) to spike Tenax TA cartridges.
  b. Use PVC tubes to attach the unridged end of each cartridge to the suction pump and then start the pump with a flow rate of 400 mL/min.
  c. Using a micropipette, add the equivalent volume of above-mentioned amounts of standard solution directly into the crimped end of each cartridge and let the pump aspire for 1 min.
    **CRITICAL:** Use each of the above-mentioned amounts in triplicates, i.e., spike three cartridges for 1 ng, three for 10 ng and so on.
  d. Inject all the cartridges spiked with the standard solution into GC-MS using the same method as described in step 2.

### Standard curve preparation and retention index calculation

**Timing: variable**

This step describes how to determine the amount of each of the compound of interest present in the pheromone through a standard curve as well as calculate their retention indices.

6. Preparing the standard curve:
  a. Integrate the areas of the GC peaks obtained for each of the calibration standards as well as the compounds of interests identified in step 3 using Agilent Mass Hunter software.
  b. Get the area values for the calibration standards in triplicates and prepare a standard curve.
    ***Note:*** For a proper standard curve, the value of the coefficient of determination (R^2^) should be above 0.9.
  c. Obtain the linear equation of the standard curve in the form of y=ax+b, where b is the y-intercept and a is the slope.
  d. Calculate the amount of the compounds of interest present in the sample from the above equation as x=y−b/a, where y is the area of the respective compounds peak.
    ***Note:*** The amount of each compound is proportional to the area of the respective GC peak.
7. Retention index calculation:
  a. Spike a Tenax TA cartridge with 10 μL of a commercial n-alkane solution and inject into GC-MS using the same method described in step 2.
  b. Obtain the retention times (RT) for each n-alkane.
  c. Compare the retention times with the same of the peaks of interest obtained in the sample and calculate the retention index according to the Kovats formula: **I**^**T**^ **= 100z+ 100 (T**_**i**_ **– T**_**z**_**)/T**_**Z+1**_ **- T**_**z**_, where T_z_<T_i_<T_Z+1_. T_i_, T_z_, and T_Z+1_ are the retention times of the compound of interest and of the bracketing n-alkanes with Z and Z+1 carbon atoms, respectively.

## Expected outcomes

**Figure 3:**
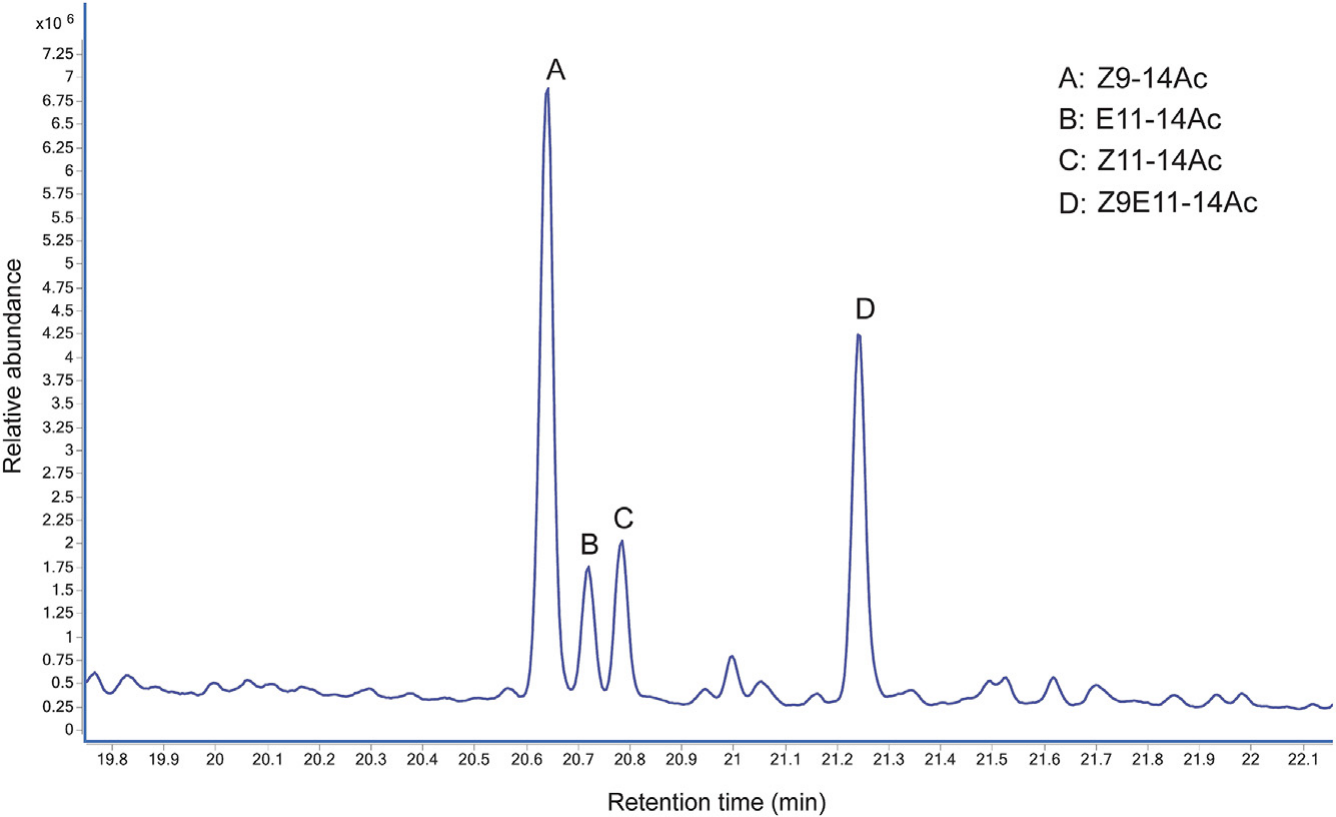
GC-MS chromatogram showing the relative abundance and retention times of the airborne pheromone components released by *Spodoptera littoralis* females Modified after Ghosh et al., 2024.^1^

**Figure 4:**
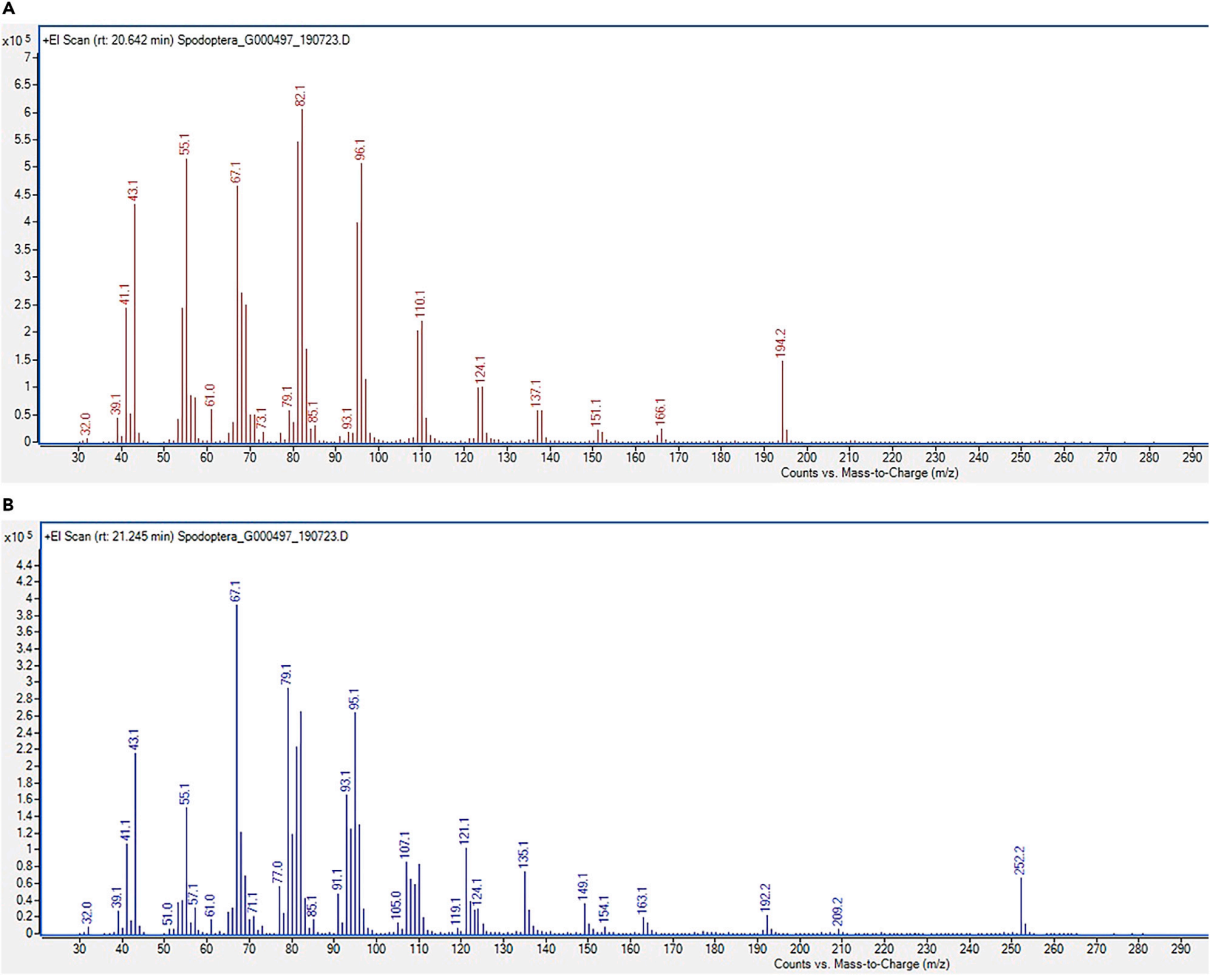
The mass spectra of two major compounds detected in the volatile pheromone of *Spodoptera littoralis* (A) Z9-14Ac. (B) Z9E11-14Ac.

In case you use *Spodoptera littoralis* as your experimental model for identifying the airborne candidate pheromone compounds released, in step 3 you should get four major peaks around the RT 20–22 min range (Figure 3). Through the NIST library, we could identify those compounds as (Z)-9 Tetradecen-1-ol Acetate (Z9-14Ac), (E)-11 Tetradecen-1-ol Acetate (E11-14Ac), (Z)-11 Tetradecen-1-ol Acetate (Z11-14Ac) and (Z, E)-9,11-Tetradecadien-1-ol Acetate (Z9E11-14Ac). As described in steps 4–6, we have injected the four calibration standards to prepare the standard curves and calculated the amount of each of these compounds present in the *S. littoralis* female pheromone (Table 1). The mass spectra of two major compounds (Z9-14Ac and Z9E11-14Ac) are given in Figure 4. We have also calculated the retention indexes of the four compounds as given in Table 1.

**Table 1:** Relative abundance of the pheromone components in the pheromone-laden air

Compound	Retention time (min)	Peak area	Amount (ng)	Retention index	Relative ratio
Z9-14Ac	20.64	1131129	84	1799	100
E11-14Ac	20.72	280178	16	1805	20
Z11-14Ac	20.78	242398	13	1809	16
Z9E11-14Ac	21.24	719456	62	1846	74

## Limitations

This protocol is not always able distinguish between the isomers present in the pheromone as their Mass Spectra are very similar.

Often the calibration standards for the compounds of interest are not available commercially, and needed to be synthesized in the lab, which is time-consuming and costly.

## Troubleshooting

### Problem 1

Scales of the moths are blocking the Tenax TA cartridge (step 1f).

### Potential solution

Add a tiny filter at the outlet of the Drechsel bottle head before connecting the cartridge to it. This should prevent the scales from going into the cartridge while allowing the passage of the air with the insect volatiles.

### Problem 2

GC-MS shows the message “cryo timeout” (step 2c).

### Potential solution

Liquid CO_2_, which is normally employed to cool down the Cooled Injection System (CIS) might be finished. So, the CIS/PVT system is unable to reach −40°C. Replace the CO_2_ cylinder with a new one to solve the issue.

### Problem 3

The GC-MS lost pressure and stopped in between two samples (step 5d).

### Potential solution

This usually happens when you use an autosampler for injecting multiple samples one after another, and the autosampler does not load one cartridge properly into the TDU. So, the TDU remains unlocked and doesn’t hold the proper N_2_ pressure (6.78 psi). Verify it and relaunch the sequence to solve the issue.

### Problem 4

The chromatogram obtained has a lot of background noise.

### Potential solution

The Tenax TA cartridge tends to adsorb all kinds of volatile organic compounds present in the air. So, make sure the air used is charcoal-filtered or use synthetic air from cylinder. It might be useful to run a blank experiment without moth for a negative control to avoid false identification of compounds.

## Resource availability

### Lead contact

Further information and requests for resources and reagents should be directed to and will be fulfilled by the lead contact, Abhishek Chatterjee (abhishek.chatterjee@inrae.fr).

### Technical contact

Questions about the technical specifics of performing the protocol should be directed to and will be answered by the technical contacts, Antonio Palazzo (antonio.palazzo12@gmail.com) and Jérémy Gévar (jeremy.gevar@inrae.fr).

### Materials availability

A sample of the moth species relevant to this study (*S. littoralis*) is available free of charge to researchers upon request to the lead contact.

### Data and code availability

This study did not generate datasets or code.

## Acknowledgments

This work was supported by 10.13039/501100022077INRAE SPE IB 2022 and 2023 grants. We thank the GC-MS facility of the EcoSens Department of INRAE Versailles for giving us access to GC-MS. We also thank Caroline Suray for helping us with the pheromone collection experiments.

## Author contributions

S.G., A.P., and A.C. designed the protocol. J.G. and P.L. helped in the experiments. S.G. and A.P. wrote the manuscript with input from A.C., J.G., and P.L. A.C. directed and supervised the project.

## Declaration of interests

The authors declare no competing interests.
