# Supplementary material for: Protocol for aerial trapping and analyses of candidate pheromone compounds released by moths via gas chromatography-mass spectrometry
Source: STAR Protoc. 2024 Sep 5;5(3):103293. doi: 10.1016/j.xpro.2024.103293 (PMC11408274; doi:10.1016/j.xpro.2024.103293)
Supplement: Document S1. Figures S1–S3 [file mmc1.pdf]

A B

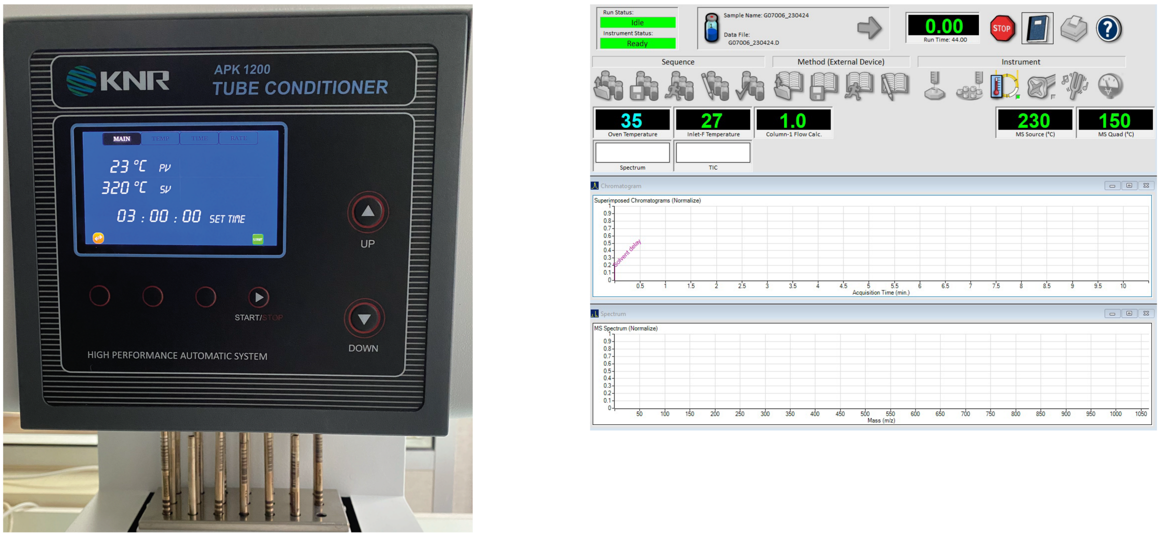

Figure S1: User interfaces and parameters to be used in this protocol, related to 'Before you begin' and Step 2. (A) Parameters of the KNR cartridge conditioner, (B) user interface of Agilent MassHunter Software.

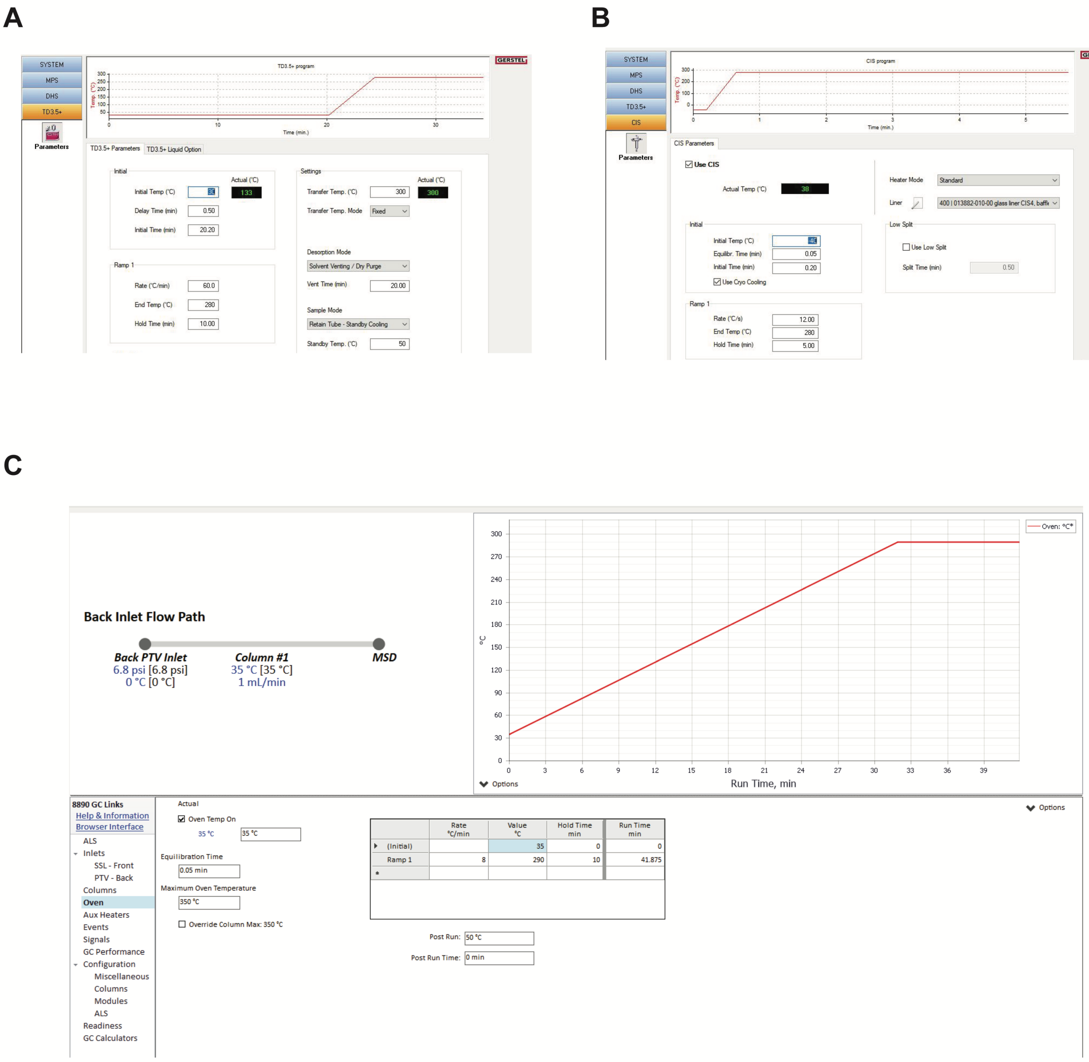

Figure S2: GC parameters during the injection of the samples, related to Step 2. (A) Parameters for Thermal Desorption Unit, (B) Parameters for Programmable Temperature Vaporizing inlet, (C) Parameters for the Oven.

A

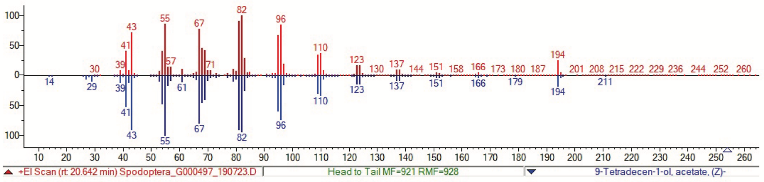

B

#	Lib.	Match	Prob. (%)	Name
⊞ 1	M	921	10.9	9-Tetradecen-1-ol, acetate, (Z)-
⊞ 2	R	919	10.0	9-Tetradecen-1-ol, acetate, (E)-
⊞ 3	R	917	9.24	cis-7-Tetradecen-1-yl acetate
⊞ 4	M	915	8.52	Z-8-Tetradecen-1-yl acetate
⊞ 5	R	904	5.84	trans-11-Tetradecenyl acetate
⊞ 6	M	903	5.62	E-9-Tetradecen-1-ol formate
⊞ 7	M	899	9.24	cis-7-Tetradecen-1-yl acetate
⊞ 8	R	897	4.41	11-Tetradecen-1-ol, acetate, (Z)-
⊞ 9	M	897	4.41	11-Tetradecen-1-ol, acetate, (Z)-

Figure S3: Identification of the pheromone component Z-9 Tetradecen-1-ol Acetate, related to Step 3. (A) Comparison of the mass spectrum with the reference spectrum, (B) Verification of the match value obtained in the NIST library.
